# Role of Melatonin in Traumatic Brain Injury and Spinal Cord Injury

**DOI:** 10.1155/2014/586270

**Published:** 2014-12-21

**Authors:** Mehar Naseem, Suhel Parvez

**Affiliations:** Department of Medical Elementology and Toxicology, Jamia Hamdard, New Delhi 110062, India

## Abstract

Brain and spinal cord are implicated in incidences of two of the most severe injuries of central nervous system (CNS). Traumatic brain injury (TBI) is a devastating neurological deficit involving primary and secondary injury cascades. The primary and secondary mechanisms include complex consequences of activation of proinflammatory cytokines, cerebral edema, upregulation of NF-*κ* *β*, disruption of blood-brain barrier (BBB), and oxidative stress. Spinal cord injury (SCI) includes primary and secondary injury cascades. Primary injury leads to secondary injury in which generation of free radicals and oxidative or nitrative damage play an important pathophysiological role. The indoleamine melatonin is a hormone secreted or synthesized by pineal gland in the brain which helps to regulate sleep and wake cycle. Melatonin has been shown to be a versatile hormone having antioxidative, antiapoptotic, neuroprotective, and anti-inflammatory properties. It has a special characteristic of crossing BBB. Melatonin has neuroprotective role in the injured part of the CNS after TBI and SCI. A number of studies have successfully shown its therapeutic value as a neuroprotective agent in the treatment of neurodegenerative diseases. Here in this review we have compiled the literature supporting consequences of CNS injuries, TBI and SCI, and the protective role of melatonin in it.

## 1. Introduction

Central nervous system (CNS) is one of the complex systems in the body which consists of brain and spinal cord. Any disease or traumatic assault may lead to the degeneration of CNS including loss of homeostasis [1]. CNS injuries constitute a major cause of morbidity and mortality include the life threatening injuries such as traumatic brain injury (TBI) and spinal cord injury (SCI) [2–4]. TBI and SCI are caused by both primary and secondary injuries influencing the cascades of cellular and molecular events which will cause further damage in the system and loss of body functions. The consequences of the secondary injury include mitochondrial dysfunction, neurotransmitter accumulation, blood-brain barrier (BBB) and blood spinal cord barrier disruption, apoptosis, excitotoxic damage, initiation of inflammatory, and immune processes which is followed by initial primary mechanical trauma [5, 6]. Secondary injury involves the production of highly reactive species, reactive oxygen species (ROS), reactive nitrogen species (RNS), or free radicals which will cause damage to protein structure, DNA, and cell membrane and leads to oxidative stress which plays a major role in the pathophysiology of CNS injury. The progression of the damage starts from the primary impact on brain or spinal cord and will continue for hours, days, and weeks after the initial mechanical insult which will result in tissue damage. This review in which two models of CNS injury will be covered will explore the role of reactive oxygen species (ROS) and current research on therapeutic role of melatonin used in the treatment of TBI and SCI.

## 2. Oxidative Stress

Oxidative stress is a phenomenon which occurs when there is an imbalance between prooxidants and antioxidants in the living system, which plays a major role in the pathophysiology of many disorders or injury. The cells of CNS have very low capacity to attenuate the effects of oxidative stress, so CNS is highly sensitive to oxidative stress and thus oxidative damage as the oxidative stress is associated with the pathogenesis of CNS injury such as stroke, TBI, and SCI.

There is a balance between the oxidants and antioxidants activities and when there is any disturbance caused by any environmental or genetic factor, excessive generation of free radicals or ROS occurs which overwhelms the endogenous antioxidant systems. Free radicals are highly reactive molecules having one or more unpaired electrons in their outermost orbits which result in their instability and make them more reactive as compared to their corresponding nonradicals. They participate in a number of reactions and form very reactive metabolites which includes hydroxyl radical (HO^∙^), hydrogen peroxide (H_2_O_2_), singlet oxygen, and peroxynitrite. Superoxide anion radicals (O_2_^∙−^) which are derived from oxygen are the most commonly occurring and one of the strongest ROS among the cellular free radicals generated in living systems [7, 8]. The superoxide radical which is considered as the “primary” ROS is capable of forming secondary ROS by reacting with other molecules. Superoxide dismutase (SOD) is a superoxide scavenging enzyme which plays the central role in amelioration of the effects of superoxide anion radicals by dismutation reaction in which it converts O_2_^∙−^ into nonradical species, singlet oxygen, and hydrogen peroxide (H_2_O_2_). H_2_O_2_ forms highly reactive hydroxyl free radical (OH^∙^) via Fenton reaction which causes damage to lipid, proteins, and DNA, which is converted into H_2_O and oxygen by an antioxidant enzyme catalase [9, 10] (Figure 3). Reactive by-products of oxygen, such as superoxide anion radical (O_2_^−^), H_2_O_2_, and the highly reactive hydroxyl radicals (^∙^OH), are formed continuously as a by-product of oxidative metabolism, catalysed by several membrane-associated respiratory chain enzymes and considered as the major source of oxidative injury in all aerobic organisms [11, 12].

ROS plays an important role as secondary messengers in many intracellular signalling pathways and also as mediators of oxidative damage and inflammation [13]. CNS has high levels of oxygen demand and unsaturated lipid content with which free radicals can easily react. Lipids are susceptible to oxidative stress and one of the products of oxidative damage to lipids is 4-hydroxy-2-nonenal (4-HNE) and acrolein and free radicals can attack directly polyunsaturated fatty acids in membranes and initiate lipid peroxidation (LPO). These features may make the CNS a target tissue for the onset and pathogenesis of a number of CNS disorders via oxygen radical production and LPO [14, 15]. It has been well documented that in the CNS the transcription factor nuclear factor erythroid-2-related factor 2 (Nrf2) which is the principal regulator of phase-2 cellular antioxidant response plays the central role in astrocyte-mediated protection of neurons from ROS [16].

## 3. Traumatic Brain Injury

TBI is devastating and a leading cause of injury related mortality and morbidity and may result in permanent functional impairment due to both primary which is caused by the initial mechanical injury and secondary injury mechanisms [1]. Primary injury is the initial traumatic brain insult, the consequences of which are immediate and irreversible disruption of neuronal cell bodies, contusion, vascular injury, and axon shearing. The primary injury also results in tissue deformation and compression, leading to seizures, respiratory depression, apnoea, ischemia, hypoxia, parenchymal inflammation, LPO, and nitric oxide production resulting in cellular injury [4]. The secondary injury which is a delayed nonmechanical insult to brain is caused by a complex cascade of metabolic, physiologic, and biochemical factors initiated by the primary insult, continuing for hours to days after injury that results in progressive tissue damage [5].

Na^+^, K^+^-ATPase is a membrane transport protein in mammalian cell membrane which plays an important role in maintaining ionic homeostasis. It is documented that any deregulation in the activity of the Na^+^, K^+^-ATPase pump might be a common feature in CNS pathologies related to ischemic conditions and TBI, since the pump is highly sensitive to reactive species and LPO and its decreased activity will increase the intracellular concentration of Ca^2+^, thus playing a synergistic role in excitotoxicity. The ischemic condition of the brain will lead to excitotoxicity which is induced by the release of excitatory amino acid neurotransmitters, particularly glutamate from neurons injured by ischemia, following which the subsequent activation of glutamate receptors resulted in a consecutive influx of Ca^2+^, Na^+^, and K^+^ into neuronal cells through these receptors. Devastating effects of increased intracellular Ca^2+^ concentrations lead to the production of free radicals such as super oxide (O_2_^−^) and hydroxyl radical (^∙^OH) and synthesis of NO peroxidation of fatty acids, which can induce xanthine pathway and ultimately to degeneration in the cellular system and cell death by various mechanisms such as overactivation of phospholipases, calpains, protein kinases, and endonucleases [17, 18].

Brain has low level of antioxidant system and high oxidative utilization (20% of the total oxygen inspired) as well as high level of polyunsaturated fatty acids (PUFA), transition metals such as iron, which is involved in the generation of the hydroxyl radical as compared to other organs which make it more susceptible to oxidative stress [19]. PUFA is very sensitive to free radicals which directly react with it and initiate LPO; during this process more radicals are formed which will disintegrate PUFA into a number of reactive products which will cause cellular damage. The deleterious effect of secondary injury to the brain revealed a number of experimental studies and oxidative stress which generates ROS one of the main cause of pathophysiologic processes after brain trauma, including mitochondrial dysfunction leading to energy failure, production of a large number of toxic and proinflammatory molecules, increase in intracranial pressure, and a subsequent decrease in cerebral perfusion leading to ischemia, astrocyte dysfunction, ATP depletion, proteolysis, and vasogenic edema [20]. Cerebral edema in TBI leads to exacerbating the severity of brain trauma by increasing intercranial pressure (ICP). Increased ICP is an important factor determining the outcomes of TBI patients and therefore it is measured. In an experimental study it was concluded that there was a significant rise in ICP in 4 and 24 h after brain injury in TBI compared to sham [21]. There are a number of studies which have been underexplored research area to cure secondary injury after TBI.

## 4. Spinal Cord Injury

SCI is a devastating event which initiates a series of complex cellular and molecular cascade events which leads to physical and social impact on individuals and society. The series of events lead to initial tissue damage in CNS. The pathophysiology of SCI is thought to include two stages: primary injury and secondary injury which is mediated by multiple injury processes including inflammation, autoimmune response, vascular events, apoptosis, free radical-induced cell death, and glutamate excitotoxicity [22]. The primary damage is restricted to the area of the mechanical insult and is characterized by ischemia. Neuroinflammation plays an important role in the pathogenesis of neuropathic pain. A major contributor to neuropathic pain in spinal cord injury is microglial activation and release of proinflammatory cytokines IL-1*β* and TNF-*α* that will enhance pain mechanism. In spite of many experimental studies, done so far or still in the process of investigation, there is no effective therapeutics which can eliminate the devastating effects of secondary damage after SCI. The severity of SCI depends on the extent of secondary damage mediated by multiple series of cellular, molecular, and biochemical reactions. The cascade of reactions includes calcium ion influx, oxygen free radical induced LPO, inflammatory reaction, autoimmune response, myelin degradation, and vascular changes (including ischemia, vasospasms, haemorrhages, and thrombosis). It also includes activation of a variety of proteases including caspases, phospholipases, endonucleases, metalloproteinases, and apoptosis which leads to the expansion of damage [23].

Inflammation acts as a key player in the pathogenesis of SCI leading to glial scar formation, neuronal loss and demyelination, and upregulation of proinflammatory cytokine TNF-*α* immediately after SCI, can enhance vascular permeability, and may cause damage to multiple organ system and finally in the neurological dysfunction or the most devastating result may come in the form of permanent loss of motor, sensory, and autonomic function due to inability of the adult mammalian nervous system to regenerate [32–34]. ROS including O_2_^−^, H_2_O_2_, OH^∙^, and peroxynitrite are believed to attack unsaturated fats, lipids, proteins, DNA and also play an important role to exacerbate the ill effects of the secondary injury. Like brain, spinal cord is also susceptible to oxidative damage because of having rich in PUFA acids [35]. Oxidative stress is one of the well-defined consequences which plays a significant role in the pathophysiology of SCI and is considered as a hallmark of secondary injury. After the mechanical insult caused to spinal cord generation of free radical superoxide (O_2_^∙−^) occurs within the first minutes and hours of injury [36].

## 5. Proposed Neuroprotective Therapies for TBI and SCI in Animal Model

Endogenously occurring hormones or other compounds such as progesterone [21, 37], estrogen [1], and lipoic acid [38] have been studied for their multifaceted role in neuroprotection. Among the endogenous compounds melatonin represents one of the best promising candidates to be studied for its role in neuroprotection (Table 1).

## 6. Biology of Melatonin

Melatonin (N-acetyl-5-methoxytryptamine), an amphiphilic molecule, was isolated by Lerner and his coworkers in 1956 in the extract of bovine pineal tissue with skin lightening properties [39, 40]. Melatonin is a methoxyindole derivative produced by extra pineal tissues and organs such as retina, Harderian gland, gut, bone marrow, platelets, astrocytes, glial cells, lymphocytes, pancreas, kidneys, and skin but predominantly by the pineal gland. It is synthesized by pinealocytes which is the main source of melatonin in blood and CNS that peaks during the night in mammals and considered as a powerful antioxidant [41]. Melatonin occurrence has been well documented in animals but its occurrence in plants has also been mentioned [42].

**Figure 1:**
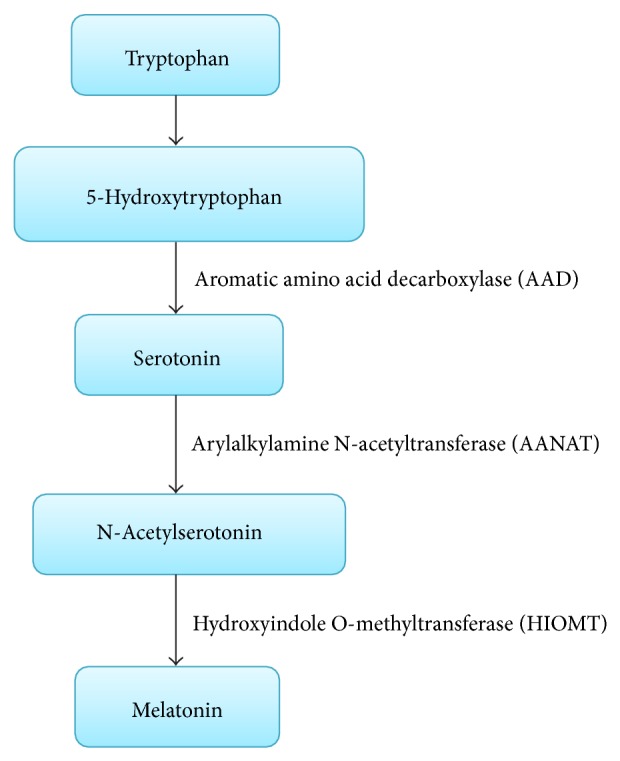
Biochemical synthesis of melatonin. Tryptophan acts as a precursor for melatonin synthesis. AAD is responsible for the conversion of 5-hydroxytryptophan into serotonin. Melatonin is formed from serotonin through a phase of reaction with the help of two enzymes, AANAT and HIOMT. Acetylation of serotonin occurs with the action of AANAT which forms N-acetylserotonin and subsequently HIOMT converts N-acetylserotonin into melatonin.

The rate of endogenous melatonin synthesis depends on the action of its precursors, the amino acid as well as a neurotransmitter serotonin, arylalkylamine N-acetyltransferase, (AANAT) and tryptophan hydroxylase (TPH) in a circadian and seasonal manner [40] (Figure 1). There are a number of studies where melatonin has been shown to regulate various physiological functions in the body such as immune enhancing, anti-inflammatory properties [43], radicals scavenger [44], traumatic CNS injury [4], modulation of human mood and behaviour [45], anticancer activity [46, 47], in oral cavity [48], against angiogenesis in breast tumors [49], sleep regulation [50].

Melatonin activity is mediated by the specific receptors in cellular membranes by two high affinity melatonin receptors, MT1 and MT2, which belong to the seven-transmembrane G-protein-coupled receptor (GPCR) superfamily and through nuclear receptors RZR/ROR. These melatonin receptors, MT1 and MT2, are primarily found in the endogenous circadian master clock suprachiasmatic nuclei (SCN) in the hypothalamus of the mammalian cells, being localized primarily to neuronal elements and in many other organs as well, coordinate the synthesis of melatonin in the pineal gland, and also participate in several neuroendocrine and physiological processes. Melatonin receptors are widely distributed in CNS as well as in the peripheral organs which acts as a lipophilic and hydrophilic molecule and able to pass the morphophysiological barriers such as the BBB. Its neuroprotective nature has also been described through the activation of these receptors but the actual mechanism is unknown.

Metabolisation of melatonin occurs by cytP-450 enzyme which metabolises it into 6-hydroxymelatonin which is followed by conjugation reaction resulting into the formation of 6-sulfatoxymelatonin [43]. In a recent experimental study it was shown that there were an increased number of MT1 receptors patients as compared to MT2 receptors where no alteration has been shown in the SCN of depressed person [51]. It also has an affinity for a third melatonin receptor, considered to represent a membrane-bound receptor (MT3), identical to the cytosolic enzyme, quinone reductase 2 (QR2) [52]. Melatonin may be used for the treatment of sleep disorders, such as insomnia as it is having hypnotic and chronobiotic properties [53]. ROS generation is a continuous phenomenon in cells and the cells have their own antioxidant system to combat this oxidative stress which is a consequence of free radicals and one of the pathophysiological causes of neurological disorders [54]. It is well documented in previous literatures that the melatonin regulates the enzymes involved in redox cycle and is a potent free radical scavenger, as its administration maintains the ratio of GSH/GSSG and LPO during aging seen in the brain mitochondria of male as well as female mice [19].

It has also been demonstrated that melatonin regulates both the electron transport chain and oxidative phosphorylation by maintaining ATP level in normal cells and also during aging. It is lipophilic in nature so it gets accumulated in the mitochondria at higher concentration and interacts with mitochondrial membrane so as to protect the brain mitochondrial membranes and mtDNA as well as electron transport chain from the damage caused by ROS/RNS by radical avoidance accomplished without the intervention of a receptor [55]. Melatonin is a broad-spectrum antioxidant and versatile as compared to its naturally occurring molecular analogs.

## 7. Melatonin: As a Neuroprotectant

The research has been in process for more than 10–12 years which has proved melatonin's beneficial effects in experimental models of neurodegenerative disorders and CNS injuries. The neuroprotective effects of melatonin appear to be mediated by the antioxidant capacity of this pineal hormone. The neuroprotective effects of melatonin may also be due to the specific interactions of its indole moiety with its receptors. Activated melatonin receptors of the brain take part in the regulation of the levels of neurotrophic factors such as brain-derived neurotrophic factor (BDNF), which has an important role in the maintenance of neuronal cells and is widely distributed in the central nervous system [56]. Increasing melatonin bioavailability decreased the severity of hemi-Parkinson conditions caused by 6-OHDA.

It has been reviewed that melatonin plays a great role in antiapoptotic activities via the inhibition of intrinsic apoptotic pathways and the activation of several signal pathways in stroke, Alzheimer's disease, Parkinson's disease (PD), Huntington's disease, and amyotrophic lateral sclerosis [57]. In an experimental study it was concluded that post-treatment process with melatonin individually or in combination with uridine can reduce posttraumatic edema in various brain regions in traumatic brain injury model [56]. In an experimental study it was evaluated that the treatment with melatonin (5 and 10 mg/kg, ip) improved the survival rate in a stroke model of mice and restored the integrity of BBB by mitigating the effect of ROS production and gp91phox cell infiltration [58]. Melatonin has been evaluated to be effective in TBI where it decreases brain edema, BBB permeability, and intercranial pressure and significantly increased superoxide dismutase (SOD) and glutathione peroxidase (GPx) activities whereas decreased malondialdehyde levels [59].

Melatonin has been shown to be protective in Parkinson's disease (PD) as it is tested in an experimental study that in combination with silymarin, it provides neuroprotection against manganese ethylenebisdithiocarbamate and 1,1′-dimethyl-4,4′-bipyridinium (paraquat) induced PD [60]. Melatonin plays a significant neuroprotective role in mitigating the cognitive impairment induced by sleep deprivation (SD) and also restores the levels of oxidative stress markers including NO and MDA as well as SOD activity [23].

## 8. Melatonin: As an Antioxidant

**Figure 2:**
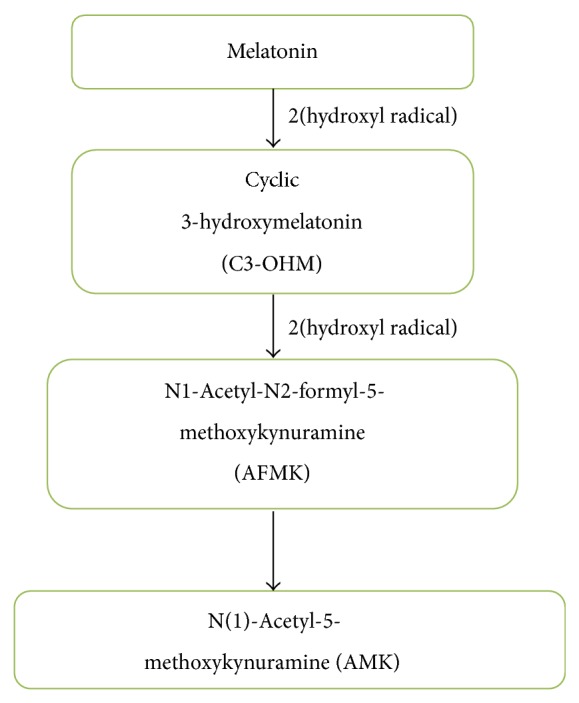
Mechanism for the formation of metabolites by oxidative pyrrole ring cleavage of melatonin forming a primary metabolite AFMK. The secondary metabolite AMK has potent radical scavenging properties as compared to AFMK, formed during the oxidation of melatonin by hydroxyl radicals (^∙^OH) as a result of pyrrole ring cleavage.

**Figure 3:**
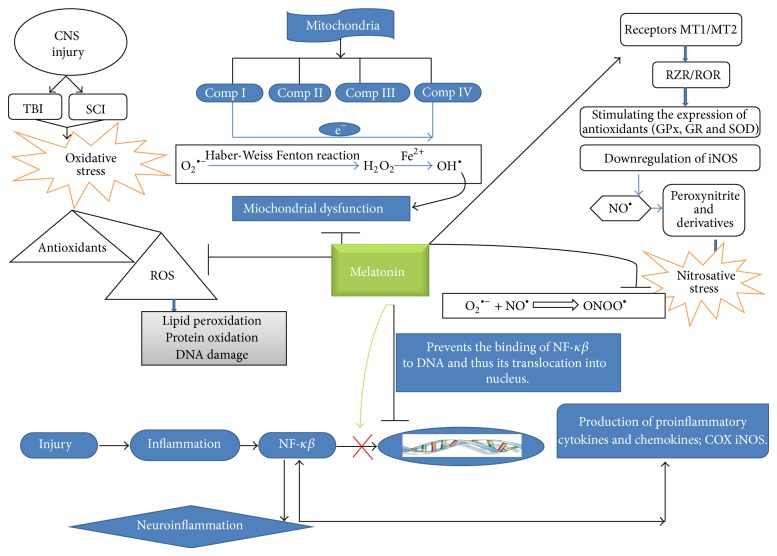
Role of melatonin in several stress conditions. CNS injury includes TBI and SCI which leads to oxidative stress which promotes ROS at a very high level compared to that of antioxidants and plays an important role in the pathogenesis of disease. ROS reacts with PUFA of lipid membranes which results in lipid peroxidation. ROS can also damage DNA and protein which leads to protein oxidation and DNA damage. Melatonin acts as a radical scavenger as it reduces the level of ROS. Fenton reaction leads to the production of hydroxyl radical OH associated with mitochondrial complexes and melatonin has metal binding capacity which may form chelating compound and reduce OH generation. Melatonin is a natural ligand for a nuclear retinoid related orphan nuclear hormone receptor superfamily RZR/ROR. Melatonin involves the regulation of expression antioxidant enzymes like GPx, GR, and SOD by involving MT1/MT2 and ROR receptors. Melatonin acts as an antinitrosative agent by reducing ONOO. CNS injury triggers cerebral inflammation which involves NF-*κβ* which is a key player in secondary injury. Here melatonin acts as an antineuroinflammatory agent and inhibits the activation of NF-*κβ* and its binding to DNA.

Melatonin as well as several of its metabolites is proved to be an effective antioxidant as its antioxidative property is also synergised by its metabolites formed from the free radical scavenging and also functions as an efficient radical scavenger [42]. Melatonin undergoes oxidation reaction resulting in the formation of cyclic 3-hydroxymelatonin (C3-OHM) which functions as a radical scavenger and scavenges two ^∙^OH resulting into the formation of N1-acetyl-N2-formyl-5-methoxykynuramine (AFMK) [61]. AFMK is also formed directly from the melatonin when melatonin reacts with H_2_O_2_. AMK (N1-acetyl-5-methoxykynuramine) may even be a potent free radical scavenger of nitric oxide (NO) compared to its precursor AFMK which is formed from AFMK via pyrrole ring cleavage [43] (Figure 2). In a recent experimental research it was concluded that oral administration of melatonin (10 mg/kg) induced a significant decrease in brain malonaldehyde (MDA) level and raised brain glutathione peroxidase activity against diazinon induced neurotoxicity [62]. It is also documented that melatonin (5 and 20 mg/kg, i.p.) was effective against the increase in reactive species and protein carbonyl levels as well as the inhibition of superoxide dismutase activity [63]. Exposure of animals to melatonin decreased LPO, restored GSH content, and mitigated the activities of CAT, SOD, and GPx in the brain from cholestatic animals. The neuroprotective effect of melatonin was shown to be more evident in the cortical and cerebral areas where it reduced the MDA content [64].

It is well documented in a number of studies that CNS is susceptible to oxidative stress or nitrosative stress caused by ROS/RNS which may cause neuronal damage if the severe effects of ROS are not suppressed or if they are not eliminated. Melatonin is an endogenously occurring hormone and is also considered as a potent antioxidant which protects the various organs of the body via its antioxidative mechanism. Melatonin is capable of scavenging a variety of ROS and RNS including hydroxyl radical (HO^∙^), superoxide anion radical (O_2_^∙−^), hydrogen peroxide (H_2_O_2_), nitric oxide (NO^∙^), and peroxynitrite anion (ONOO^−^) and at the same time it also activates several antioxidant enzymes, including glutathione peroxidase, superoxide dismutase, and catalase. From this it can be concluded that melatonin has been used as an antioxidant from many years and several of its metabolites are also able to protect cells from oxidative damage caused by reactive species and it amplifies the antioxidant capacity of melatonin.

## 9. Neuroprotective Mechanisms of Melatonin in TBI

### 9.1. Reduced Inflammation

Inflammation after the mechanical insult exacerbates the devastating effects of CNS injury. CNS injury such as SCI produces a marked neuroinflammatory response characterized by influx of monocytes such as macrophages and neutrophils, activation of microglial cells and astrocytes which contribute to the secondary pathological and inflammatory response, upregulation of proinflammatory cytokines such as IL-1*α*, IL-1*β*, IL-6, TNF-*α*, interferon-Y, and intracellular adhesion molecules (ICAM-1), and also contributed to neuroinflammation which have contributed to facilitate CNS inflammatory responses by inducing expression of ROS such as superoxide radical and nitric oxide (NO) and chemokines, which inhibit the process of neurogeneration and also exacerbate cerebral damage, inflammation of which has a great role in the pathogenesis of secondary injury in TBI [65, 66]. It has been reported that melatonin has a potential role in deactivating the release of proinflammatory cytokines in TBI [43].

### 9.2. Reduced Edema

Cerebral edema is the critical factor involved in the pathogenesis of secondary injury in TBI. Different reasons have been suggested for the occurrence of edema in TBI such as swelling of damaged cells and injury to blood vessels that forces fluid to enter the various regions of brain and blood-brain barrier breakdown and increase of endothelium permeability and vascular permeability. Cerebral edema can be classified into two main categories, namely, cytotoxic edema (closed barrier) or vasogenic edema (open barrier). Vasogenic brain edema is characterized by the structural and functional disruption of the blood-brain barrier and involves accumulation of proteins due to increased permeability of endothelial cells to albumin, dextran, and so forth, whereas cytotoxic edema is the result of cellular uptake and cell swelling without interfering the integrity of blood-brain barrier. Cerebral edema when left untreated results in an increase in intracranial pressure that may lead to limiting cerebral perfusion, oxygenation of the tissue, distortion, herniation, and even death [67]. Previous studies have shown that administration of melatonin after TBI in albino N-Mary rats model decreases brain edema, BBB permeability, and ICP at 72 h after TBI at low as well as high dose [59]. In an another study administration of melatonin (200 mg/kg), both individually and in combination with uridine, reduced posttraumatic edema in various brain regions of male Sprague-Dawley rat model of TBI [56].

### 9.3. Regulation of NF-*κ* *β*

It has been well documented in a previous study that transcription factor (NF-*κ* *β*) has diverse function in regulating synaptic transmission and plasticity, spatial memory function, and development and increased activation of which has been seen after different pathological conditions including traumatic brain injury, ischemia [68]. The mRNA expressions of NF-*κ* *β* were shown to be upregulated remarkably following TBI [35]. Efficacy of melatonin is investigated in alteration of the molecular changes that occur as a result of closed head injury (CHI). Melatonin totally blocks the late-phase activation of NF-*κ* *β* and decreases that of AP-1 to half the basal level. An effective dose of melatonin 5 mg/kg has shown the significant effect by mitigating damage [69].

### 9.4. Regulation of BBB Integrity

TBI can result in brain and BBB disruption resulting into increased BBB permeability. Disruption of the BBB may lead to astrocytic dysfunction, inflammation-related mechanisms, and permeability to endogenous proteins which may cause brain dysfunction [70, 71]. In a clinical study it was quantified that there was a lasting focal increase in BBB permeability in up to 70% of TBI patients [72]. In a recent study it was documented that melatonin administration in both low and high doses can significantly decrease blood-brain barrier permeability at 72 h after TBI [59].

### 9.5. Reduced Oxidative Damage

Oxidative stress plays an important role in the pathogenesis of secondary injury following TBI. It was evaluated in a study that the brain GSH content decreased significantly and TBARS level which is the by-product of LPO increased significantly, resulting into enhanced LPO following TBI [20]. LPO is the process in which free radicals steal electrons from the lipids and cause degradation of lipids and ultimately cell membrane. Melatonin has been shown to be a good neuroprotectant in various studies by suppressing oxidative stress markers and by upregulating endogenous antioxidants maybe due to its lipid soluble characteristic due to which it easily crosses morphophysiological barriers which make melatonin an ideal antioxidant and its antioxidative action is independent of receptors [52]. Recently it has been found that daily administration of melatonin after TBI increases SOD and GPX activity and decreases the MDA level significantly within 72 h and thus protected cerebral tissue against oxidative stress [59].

## 10. Neuroprotective Role of Melatonin in SCI

### 10.1. Reduced Inflammation

In an experimental literature it was demonstrated that proinflammatory cytokines such as interleukin 1 (IL-1*α*) and IL-1*β* are activated within the few hours in SCI [73]. It has been demonstrated that IL-6 plays a great role in the pathogenesis of proinflammatory damage after SCI and TNF-*α* which is having a significant role in immune and vascular responses and is released early after the inflammation plays a pivotal role in the process of inflammation by increasing the number of inflammatory and immune cells to the injured site [33]. Activation of NF-*κ* *β* signalling pathway has been shown to be important for the induction of inflammation and a pathophysiologic cause of spinal cord inflammatory response after injury and it also takes part in the generation of ROS and prostaglandins (COX and iNOS) which act to synergise the effects of inflammation [33]. In an experimental literature it was concluded that the levels of the p65 subunit protein in the nuclear fractions of the spinal cord tissue were significantly increased at 24 h after SCI [28]. In response to the inflammation proinflammatory cytokines, the invasion of neutrophils and CNS macrophages is shown to be greater in spinal cord [74]. Melatonin which is an endogenous hormone produced by tryptophan shows its potency of neuroprotection, suppresses the expression of NF-*κ* *β* regulated adhesion molecules, and reduces the production of proinflammatory cytokines [75]. If we consider the findings during the past few years, then there are strong evidences of this hormone that it could be a key player in preventing the severity of secondary damage which could lead to the neuronal death, by improving the recovery function in injured neuronal cells [76]. It has been well documented that melatonin is also protective against the devastating effects of inflammation by reduction of the production of several proinflammatory cytokines thus preventing neurons which results from the various neurological disorders [77]. In another study it was concluded that melatonin treatment was effective in reducing the activation of inflammation and tissue injury in an animal model of spinal cord injury [78].

### 10.2. Regulation of iNOS

Nitric oxide (NO) which is a neurotransmitter, as well as a free radical, is able to form peroxynitrite (ONOO^−^) by reacting with superoxide forming a more potent and devastating oxidant [79]. NOS exists in three genetically different isoforms: neuronal NOS (nNOS, NOSI), located within central and peripheral neurons and some other types of cell, inducible NOS (iNOS, NOS II) which is Ca^2+^-calmodulin independent enzyme associated mainly with macrophages and microglial cells, and endothelial NOS (eNOS, NOS III), expressed within vessel endothelia [30, 80, 81]. Nitric oxide is produced from L-arginine-NO pathway by the NO synthase involving NADPH and O_2_ and NO is involved in several cellular functions, such as neurotransmission, regulation of vascular tone, apoptosis, and immune system [82]. In an experimental study it was investigated that all SCI groups showed an upregulated iNOS mRNA expression in damaged regions of the spinal cord compared with animals with an intact spinal cord and highest expression of iNOS mRNA was detected at 3 days after injury and it was shown that administration of melatonin 10 mg/kg body weight significantly diminished iNOS expression and NO production [30].

### 10.3. Regulation of MAPK

Mitogen-activated protein kinases (MAPKs) which play important roles in cell signalling and gene expression consist of the extracellular signal-regulated protein kinase (ERK), c-Jun N-terminal kinase (JNK), and p38 pathways, in which p38 MAPK is regarded as a stress-induced kinase [83, 84]. In a study a significant increase in phosphorylated-pERK1/2 levels was observed in mice model of SCI [85]. In an experimental study it was reported that 24 h postspinal cord trauma a dose of 50 mg/kg melatonin reduced activation of MAPKs p38, JNK, and ERK1/2 [86].

### 10.4. Reduced Oxidative Stress

Oxidative stress by free radical generation and LPO are the hallmarks of secondary injury caused by SCI. In recent years, much attention has been focused on oxidative stress cascade leading to secondary injury after SCI because inhibition of the deleterious effects of it is thought to be one of the main mechanisms of therapeutic interventions [87]. ROS and RNS are the free radicals which cause devastating effects in biological system [88]. Recently it has been shown that MDA content significantly increased at 1 day after injury and reached a peak value at 7 days in the SCI group and SOD activity was significantly reduced in the SCI group at 1 day. From 1 to 14 days, SOD activity was significantly reduced in the SCI group [29]. Melatonin and its metabolic derivatives have been documented to be an effective direct free radical scavenger and also stimulate the activities of several endogenous antioxidative enzymes [89]. In a SCI model of Wistar albino rats, MDA level was found to be increased in spinal cord in SCI group of animals while treatment with melatonin attenuated the elevated levels in MDA level in spinal cord tissue. SCI has also been shown to cause a significant decline in GSH level whereas treatment with melatonin (10 mg/kg) was able to restore the decreased level of GSH level in spinal cord tissue [90].

## 11. Adverse Effects of Melatonin

Melatonin is a hormone which is the main secretory product of pineal gland and has been shown to exhibit antioxidant properties by increasing intracellular antioxidants activity or acting as a direct radical scavenger. However recent evidences indicate that melatonin is also able to generate intracellular ROS [91, 92]. The deleterious effect of melatonin is not much explored even though it can cross cell membranes due to its amphilicity as it has been shown to decrease intracellular free thiols by melatonin [93]. It has been explored that the activity of intracellular activity GSH reduced due to melatonin treatment in U937 cells which is correlated to its ability to produce intracellular ROS after a certain time period [91]. ROS may be involved in inducing apoptosis as seen in various studies and thus act as a prooxidant as well as a proapoptotic factor for causing apoptosis [94, 95]. Various studies on antiapoptotic activity of melatonin are reported in which the administration of exogenous melatonin has been shown to be effective in preventing normal cells from apoptosis in* in vitro* [96, 97] as well as* in vivo* [98]. But research reports also indicate melatonin has been reported to have proapoptotic and cytotoxic features in several cancer cells including the human myeloid cell line HL-60 [99–101]. Its cytotoxicity in Caco-2 cells in certain concentration range has been well proved in a recent study [102]. In a study it was shown that melatonin in Jurkat cells was responsible for prooxidant activity [103].

## 12. Protective Role of Melatonin in Human

Melatonin has been shown to be effective in neuroprotection in various animal models [104–107]. However a number of scientific literatures have also investigated the neuroprotective role of melatonin in human. Melatonin which is the main secretory body of pineal gland is believed to be an important part in sleep/wake cycle regulation and also the sleep disorder in old age people [108]. The biological clock in the hypothalamic suprachiasmatic nucleus (SCN) regulates melatonin secretion by the pineal gland as well as circadian rhythm regulation [109]. Endogenous melatonin as well as exogenous melatonin has been seen to be effective in combating oxidative stress in brain. It has long been well reported that the level of endogenous CSF melatonin increases in TBI patients to suppress the level of oxidants which has a direct link in the pathogenesis of brain injury [110]. In a double blind study melatonin seemed to be effective in treating Alzheimer type dementia (ATD) which is a progressive fatal neurodegenerative [109]. Delirium is a common neuropsychiatric condition which has shown to be decreased by exogenous supplementation of melatonin in elderly patients [108].

In conclusion, melatonin has been documented to be a potent neuroprotective agent in rodent models of traumatic brain injury and spinal cord injury. Melatonin, which is secreted by the pineal gland, is a powerful scavenger of reactive oxygen species. Additionally, melatonin is able to increase the activity and expression of antioxidant enzyme activity under both physiological conditions of elevated oxidative stress. Its inherent property of crossing the blood-brain barrier makes it more effective in mediating neuroprotection.

## Figures and Tables

**Table 1:** Proposed neuroprotective therapies for TBI and SCI in rodent models.

Model	Nutraceuticals	Dose	Results/effects
**TBI**			
(1) Mice	Minocycline	90 mg/kg at 5 min, 45 mg/kg at 3 h and 9 h after TBI.	Minocycline was able to attenuate the memory impairment in an effective and lasting manner [24].
(2) Mice	Minozac	5 mg/kg	Minozac attenuates acute increase in proinflammatory cytokine and chemokine levels and reduces astrocyte activation and the longer term neurologic injury [25].
(3) Rat	(−)-Epigallocatechin-3-gallate	0.1% (w/v) in drinking water	EGCG exposure before and after TBI decreased DNA damage and LPO levels and neuronal cell and NSC apoptosis around the damaged area following TBI at 1 day, 4 days, and 7 days [26].
(4) Rat	Alpha lipoic acid	100 mg/kg, *p.o. *	It reduced LPO, suppressing the MPO enzyme activity increased, Na^+^, K^+^-ATPase activity. It reduces edema formation and reduces BBB permeability and thereby preserves neuronal damage [20].
(5) Rat	Caffeic acid phenethyl ester	10 *μ*mol/kg	Decreased the elevated MDA levels, also significantly increased the reduced antioxidant enzyme SOD and GPx activities, reduced the immunoreactivity of degenerating neurons, and inhibited apoptotic cell death by downregulating caspase 3 [27].

**SCI**			
(1) Mice	Green tea extract	25 mg/kg, *i.p. *	Reduced upregulation of TNF-*α* or IL-1*β*, suppresses NF-*κ* *β* activation, and attenuates the expression of iNOS, nitrotyrosine, and poli (ADP-ribosio) synthetase (PARS) and neutrophilic infiltration was markedly reduced and ameliorated the recovery of limb function [28].
(2) Rat	Quercetin in combination with methylprednisolone (MP) and specific p38MAPK inhibitor SB203580.	0.2 mg/kg per day	Inhibited increases in phosphorylated-p38MAPK (p-p38MAPK) and iNOS expression and reduced the rate of iNOS-positive cells in rats with SCI, reduced MDA content, and increased SOD activity in SCI rats [29].
(3) Rat	Melatonin was administered in combination with exercise.	10 mg/kg	Significant increase in hind limb movement, reducing NO production and motor neuron degeneration, reduced level of iNOS mRNA [30].
(4) Rat	17*β*-Estradiol	100, 300, or 600 *μ*g/kg	Reduced oligodendrocyte cell death via inhibition of RhoA and JNK3 activation and axon loss [31].
